# Knowledge of AIDS and HIV transmission among drug users in Rio de Janeiro, Brazil

**DOI:** 10.1186/1477-7517-8-5

**Published:** 2011-02-15

**Authors:** Neilane Bertoni, Merril Singer, Cosme MFP Silva, Scott Clair, Monica Malta, Francisco I Bastos

**Affiliations:** 1 Institute for Scientific and Technological Information and Communication in Health (LIS/ICICT). Oswaldo Cruz Foundation, Rio de Janeiro, Brazil; 2 Sergio Arouca School of Public Health (DCS/ENSP), Oswaldo Cruz Foundation, Rio de Janeiro, Brazil; 3 Department of Anthropology, University of Connecticut, Storrs, Connecticut, USA; 4 Partnerships in Prevention Science Institute, Iowa State University, Ames, Iowa, USA; 5 Fulbright/CAPES Visiting Researcher at Brown University, Providence, RI, USA

## Abstract

**Background:**

Proper knowledge of HIV transmission is not enough for people to adopt protective behaviors, but deficits in this information may increase HIV/AIDS vulnerability.

**Objective:**

To assess drug users' knowledge of HIV/AIDS and the possible association between knowledge and HIV testing.

**Methods:**

A Cross-sectional study conducted in 2006/7 with a convenience sample of 295 illicit drug users in Rio de Janeiro, assessing knowledge on AIDS/HIV transmission and its relationship with HIV testing. Information from 108 randomly selected drug users who received an educational intervention using cards illustrating situations potentially associated with HIV transmission were assessed using Multidimensional Scaling (MDS).

**Results:**

Almost 40% of drug users reported having never used condoms and more than 60% reported not using condoms under the influence of substances. Most drug users (80.6%) correctly answered that condoms make sex safer, but incorrect beliefs are still common (e.g. nearly 44% believed HIV can be transmitted through saliva and 55% reported that HIV infection can be transmitted by sharing toothbrushes), with significant differences between drug users who had and who had not been tested for HIV. MDS showed queries on vaginal/anal sex and sharing syringes/needles were classified in the same set as effective modes of HIV transmission. The event that was further away from this core of properly perceived risks referred to blood donation, perceived as risky. Other items were found to be dispersed, suggesting inchoate beliefs on transmission modes.

**Conclusions:**

Drug users have an increased HIV infection vulnerability compared to the general population, this specific population expressed relevant doubts about HIV transmission, as well as high levels of risky behavior. Moreover, the findings suggest that possessing inaccurate HIV/AIDS knowledge may be a barrier to timely HIV testing. Interventions should be tailored to such specific characteristics.

## Background

According to the United Nations General Assembly Special Session on HIV/AIDS' (UNGASS) indicators used for monitoring and evaluating HIV/AIDS policies [1], the Brazilian population has one of the highest levels of accurate knowledge about HIV transmission modes worldwide [2]. However, as pointed out in this same study, there are relevant differences between various regions of the country and different social strata regarding the proportion of interviewees who have accurate information about the main modes of transmission of HIV/AIDS. These disparities seem to reflect the deep regional and sociodemographic disparities reported in Brazil based on a wide set of socioeconomic and demographic indicators [3]. Lower levels of correct answers (an indicator related to (im)proper knowledge on HIV/AIDS transmission, as defined by the Brazilian Ministry of Health in (http://sistemas.aids.gov.br/monitoraids/) have been found among individuals with lower socioeconomic status, as well as individuals with greater frequency of unsafe sexual practices [4].

Drug users - according to studies conducted both in Brazil and in several other countries - primarily belong to the poorest social strata and are less educated compared to the general population [5]. Most of them are young men, are sexually active, and have inconsistent condom use [6-8]. The combination of these factors makes this population a key one in the spread of different sexually (and/or blood-borne infections in the case of unsafe injection practices by injecting drug users) transmitted infections.

Authors such as Farmer [9,10] and Singer et al. [11] have shown that the risk for sexually transmitted infections among individuals from the poorest social strata is augmented compared to affluent strata, due to structural factors, such as, barriers to obtaining condoms and sterile syringes/needles (essential resources for prevention) due to cost, legal restriction or stigma, difficulties in understanding information on prevention and treatment not tailored to their literacy levels/personal and social values and attitudes, and their marginal condition in society.

Accurate knowledge on HIV/AIDS is a necessary; but, by no means sufficient condition for the consistent adoption of protective behaviors. However, the lack of such information contributes to an increased vulnerability to HIV/AIDS in many contexts, including Brazil [12]. Moreover, lack of correct information about pathways of HIV transmission may contribute to fewer people being tested, to misperceptions about one's level of risk, and to increased likelihood of AIDS optimism, denial, and stigmatization, among other adverse psychosocial influences [13].

In Brazil, in 2005, 33.6% of the urban population had been tested for HIV. However, excluding blood donation screening [14] and prenatal care routine tests, this proportion is reduced to 20.8% of the urban population [15]. This is a relatively high proportion compared to other low and middle-income countries, although less than optimal according to the standards defined by the Brazilian Ministry of Health vis-à-vis the key UNGASS indicators [16]. Among drug users, testing may be even more infrequent, as Brazilian populations with lower income and education level - common characteristics of street drug users - have lower rates for HIV testing [15], as well as other risk-enhancing behavioral, attitudinal and social features discussed in previous publications of our research group [17,18].

In a scenario of social inequities and with a concentrated AIDS epidemic, as is the case in Brazil [19], it is necessary to know the specificities of vulnerable population groups to tailor prevention, management and care to address their knowledge, attitudes and practices regarding HIV/AIDS.

This study describes and assesses the knowledge of HIV/AIDS found in a sample of drug users in the municipality of Rio de Janeiro, Brazil, using two different methodologies, as well as explores the putative association between HIV/AIDS knowledge and HIV testing.

## Methods

Data analyzed in this study refer to the baseline survey of the project "Assessing HIV Oral Testing of Brazilian Drug Users" performed by the Oswaldo Cruz Foundation in partnership with Iowa State University and the Hispanic Health Council, funded by the National Institute on Drug Abuse (NIDA [details in [18]]. Baseline data collection was conducted between May 2006 and April 2007 with a non-probabilistic sample of 295 drug users recruited in two heath services located in the municipality of Rio de Janeiro downtown and easily accessible by public transportation. These two health centers, located one block from each other, are a dedicated public center for people who misuse alcohol and drugs and a not-for-profit facility open to the community and offering treatment at no cost for any condition, including alcohol and drug misuse. Eligibility criteria were: ages between 18 and 65 years, having used drugs (other or in addition to alcohol, tobacco, and marijuana) in the last 30 days, and to not be engaged in treatment for drug abuse in the last 30 days.

The minimum sample size was defined in advance after the calculation of statistical power of hypothetical multivariable analyses that took into account the four aims of the original proposal, including the main aim comprising the evaluation of the differences between individuals that received a standard intervention (verbal counseling on sexuality transmitted infections/STIs/AIDS) from those who received a comprehensive educational intervention: i.e. the standard intervention supplemented by the use of cards with illustrations depicting "possible" methods of HIV transmission. Assuming a hypothetical situation of four key covariates (age, gender, ethnicity, and recruitment location), a single predictor (standard intervention vs. comprehensive education intervention using cards) and a single dependent variable ("changes in knowledge scores about HIV transmission through saliva"), and assuming that these covariates correspond to 5% of the total variance related to the knowledge on HIV transmission through saliva, we would have statistical power of 0.81 using a minimal sample of 210 individuals. Furthermore, we would have statistical power of 0.85 to detect a change of 0.04 in R^2 ^for a single predictor on the nature of the intervention. The three other aims, including the assessment of knowledge on HIV/AIDS versus testing, as explored in the current paper, required a smaller sample size compared to the aim associated with behavioral change, which was taken here as a benchmark, as the most strict one.

After signing the consent form, a questionnaire was administered to all participants, comprising the following twelve sections: 1) Sociodemographic information; 2) Behavior and practices relating to drug use; 3) Sexual behavior and practices; 4) HIV testing; 5) Barriers to HIV testing; 6) Facilitators (and barriers) to accessing health services; 7) Knowledge about the most vulnerable behaviors for HIV/AIDS infection; 8) Stigma associated with HIV; 9) Knowledge about hepatitis; 10) Depression; 11) Self-esteem; and 12) Locus of control ("self-control").

Questionnaires used Teleform^® ^scannable forms. The acceptability and comprehensibility of the questionnaire was previously piloted with 50 interviewees.

Initially, an exploratory analysis of socio-demographic characteristics, drug consumption patterns, and sexual behaviors was carried out. Knowledge on AIDS and HIV transmission modes was evaluated based on the "AIDS Risk Behavior Knowledge Scale," originally developed by Kelley and colleagues [20], and updated for our study based on current research findings on HIV/AIDS, as defined by the PCAP (Survey on Knowledge, Attitudes and Practices of the Brazilian Population) surveys carried out in Brazil by the BMoH every 4-5 years [2]. Individuals were asked to answer if each one of the statements/items on AIDS and HIV transmission was true, false, or if they didn't know the correct answer. In our analysis, an individual's answers were dichotomized as either "correct" or "incorrect" (incorrect + "don't know") based on the consensus about the ways HIV is actually transmitted, as defined by the World Health Organization (WHO)/The Joint United Nations Programme on HIV/AIDS (UNAIDS) and regularly used in the abovementioned PCAP surveys. Comparisons were done on an item by item basis, using Chi-square tests, between the group of participants who had ever been tested for HIV and the group that never had been tested. Analyses were performed using STATA v.9.

The Orasure^® ^Saliva Test was used to determine the HIV status of participants. No participants refused to be tested. All participants received standard pre-test counseling (verbally) about STIs/AIDS. A subsample of 144 drug users was randomly selected to receive an additional preventive intervention, consisting of giving each participant a set of 13 cards with pictures representing different types of interaction, including both direct and indirect forms of interpersonal contact, that were potential routes of HIV transmission. The direct interaction pictures included two people: kissing, using the same toothbrush, drinking out of the same glass, drinking out of the same bottle, sharing a cigarette, using the same bathroom, shaking hands, sharing injected and inhaled drugs, and having vaginal and anal sex. The indirect interaction pictures included a person being bit by a mosquito and a person donating blood.

After observing each picture, participants were asked to sort the pictures into two piles, one with illustrations of behaviors that could transmit HIV, and one with behaviors that could not lead to the transmission of HIV. The answers were recorded; and if the participant put any pictures into the wrong pile (e.g., put the mosquito picture into the HIV transmission pile or the card depicting syringe sharing into the non-transmission pile), s/he received a detailed explanation of current scientific understanding of the potential for the behavior in question to transmit HIV at the end of the pile sort. This exercise produced a final sample of 108 individuals, after cases with missing data were removed.

Pile sort data on participants' HIV transmission knowledge was analyzed using Multidimensional Scaling (MDS), a statistical method which, using a perceptual map, represents spatially the similarities and dissimilarities of a set of elements. In the present study, the perceptual map that was produced reflects the positions in space of the icons corresponding to the ways HIV can be transmitted (or not). In general, this method defines "proximity" vs. "distance" - depicted as visual representations of how similar or different two objects are based on either objective criteria or the perception of the research respondents [21]. MDS was chosen based on our desire to measure not only the knowledge of every single participant about a given behavior, but also to identify shared perceptions and evaluative dimensions of subgroups of individuals from the sample [22]. Multidimensional scaling (MDS) is a method aimed at displaying statistical information in a low-dimension geometrical space as a clearly discernable visual presentation, used for assessing contrasts and similarities of different conceptual categories. Whatever kind of relation between a pair of categories that can be translated into a proximity measure, or conversely into a dissimilarity measure, can be considered as possible input for multidimensional scaling. To assess fitness, the Stress-1 was used.

Stress-1 corresponds to the proportion of variation of original distances in relation to the predicted distances; thus, the smaller Stress-1 is, the closer to optimal fitness. This analysis was performed with SPSS 16.0.

The agreement between the responses of individuals who received the educational intervention using cards with their answers in the face-to-face interview using the questionnaire also was assessed. This final analysis was limited to four items, shared by both methods (questionnaire and cards). These items comprise: transmission through mosquito bites, blood donation, shared use of public bathrooms, and the shared use of toothbrushes.

This study was approved by the IRBs of Iowa State University (IRB ID No.: 03-824, March 15, 2004), IPEC/FIOCRUZ (Prot. n° 0003.1.011.009-04, May 10, 2004), and CONEP/CNS/MS (Registration n° 10332, September 24, 2004).

## Results

Of the 295 individuals who participated in the interview, 227 (77.0%) were male. Most were single (56.8%) and self-identified as black or biracial (41.8 and 32.7%, respectively). Few participants (4.1%) reported being homeless at the moment they were interviewed. The median age was 29 years, and the median education level was 8 years of formal education.

About 20% reported no income in the last 30 days, and 30% reported to have received less than a minimum Brazilian monthly wage during the same period (R$300). The median income in the last 6 months was R$350 per month (roughly equivalent to US$200). However, 25% of the respondents said they had spent more than R$150 per week purchasing illicit drugs (Table 1).

**Table 1:** Socio-demographic characteristics of the 295 participants of the project "Assessing HIV Oral Testing of Brazilian Drug Users".

Variables	n	%
Gender		
Male	227	77.0
Female	68	23.0
Marital status (N = 294)		
Single	167	56.8
Married/Living together	90	30.6
Separated/Divorced	34	11.6
Widowed	3	1.0
Homeless		
Yes	12	4.1
No	281	95.3
Don't know/Not sure	2	0.7
Race/Skin color (N = 294)		
White	61	20.8
Black	123	41.8
Brown	96	32.7
Indian	3	1.0
Other	11	3.7
Income in the last 30 days (N = 293)		
None	59	20.1
< R$300	91	31.1
R$ 300 to R$ 599	93	31.7
R$ 600 to R$ 1.199	33	11.3
R$ 1.200 to R$ 1.799	13	4.4
R$ 1.800 or more	3	1.0
Don't know/Not sure	1	0.3

Continuous variables	median (IQR*)	

Age	29 (23; 40)	
Years of education	8 (6; 10)	
Median income in the last 6 monts (in Reais) (N = 294)	350 (175; 500)	
Spent on drugs in the last week (in Reais) (N = 287)	50 (20; 150)	

Rio de Janeiro, 2006

* IQR: Interquartile range. Denotes Quartile 1 (25%) and Quartile 3 (75%)

The drugs the interviewees most frequently reported to have ever consumed were alcohol, marijuana, and sniffed cocaine (97.6%, 91.5% and 67.5%, respectively). Having ever smoked crack was reported by 19.3% of participants. This latter finding is consistent with the fact that the average age of first use of crack was relatively high (25 years), compared to the first use of alcohol (14 years) or marijuana (17 years). Inhalant use was reported by 61.0% of respondents, reflecting the continued popularity of this form of drug use in Brazil. Synthetic drugs use, such as ecstasy and LSD, was reported by 15.9% and 7.5% of interviewees, respectively. Injected cocaine was reported by 7.8% of the participants (Table 2).

**Table 2:** Drug used by the 295 participants of the project "Assessing HIV Oral Testing of Brazilian Drug Users".

	Use in life		Age of first time drug use	
	**n**	**%**	**mean**	**standard-deviation**

Alcohol	287	97.6^1^	14.6	3.5
Marijuana	270	91.5	17.1	5.6
Crack	57	19.3	25.2^2^	9.8
Snorted cocaine	199	67.5	18.4	5.9
Injected cocaine	23	7.8	19.1	3.9
Snorted heroin	15	5.1	20.2^2^	4.6
Injected heroin	6	2.0	18.6^2^	4.4
Speedball (cocaine and heroin)	8	2.7	20.3	5.7
Methamphetamine/amphetamine	33	11.2	19.6^2^	5.9
Anabolic steroids	31	10.5	21.1^2^	5.7
Ecstasy	47	15.9	21.7^2^	6.6
Inhalants	180	61.0	19.3^2^	7.9
LSD	22	7.5^1^	21.4	5.4
Barbituates (non-Rx)	35	11.9	26.8^3^	15.1
Benzodiazepines (non-Rx)	25	8.5	24.1^4^	8.0

Rio de Janeiro, 2006

^1 ^N = 294 ^2 ^1 missing ^3 ^3 missing ^4 ^5 missing

The reported substance use patterns may be underestimated, since 30.8% of drug users reported having ever been in treatment for drug addiction, and 74.7% of those that have been in treatment were in treatment in the last 6 months (data not shown in table), suggesting more intense consumption than that reported by the interviewees or a pattern of use that has been perceived by the interviewees and/or their families as dysfunctional (compulsory treatment mandated by courts use a different network of institutions, not assessed by the present study).

Use of drugs can modulate individuals' attitudes and behaviors. Many participants reported having ever had sex with unknown partners (65.8%) or not using condoms (61.1%) when under the influence of drugs (Table 3). A relatively high (49.4%) proportion of interviewees reported to have had at least 2 different partners in the last 30 days.

**Table 3:** Risky sexual behavior potentially associated with the use of drugs of 295 participants of the project "Assessing HIV Oral Testing of Brazilian Drug Users".

Variables	n	%
**Number of sexual partners in the last 30 days (N = 295)**		
None	28	9.5
One	121	41.0
2 to 4	109	36.9
5 or more	37	12.5
**Frequency of condom use (N = 267)**		
Never	103	38.6
Less than half the time	59	22.1
Half the time	21	7.9
More than half the time	30	11.2
Alwasys	54	20.2
**Main reason for do not use condom (N = 211)**		
Don't have one	9	4.3
Don't like to use	78	37.0
Partner don't want to use	9	4.3
Caught by surprise	23	10.9
Trust in partner	67	31.8
For (partner) pregnancy	2	1.0
Under drug effect	19	9.0
Other reason	4	1.9
**Sexual identity (N = 294)**		
Heterosexual	285	96.9
Homosexual/Bisexual	5	1.7
Don't know/Refused to answer	4	1.4
**Had sexual relation with an unknow person due to be under the influence of drug**	194	65.8
**Didn't use condom in sexual relation due to be under the influence of drug (N = 293)**	179	61.1

Rio de Janeiro, 2006.

Almost 40% of participants reported never using condoms and 22.1% had used them in less than half of their sexual intercourses. Only 20.2% of participants reported using condoms consistently in every sexual relations, irrespectively of the nature of the relationship (Table 3).

The main reason mentioned for not using condom was that they didn't like to use them (37.0%). Less than one-third mentioned they trusted their partners (31.8%). Despite the fact most interviewees mentioned attitudinal and behavioral changes under the influence of drugs, only 9.0% explicitly said that this was the main reason for not using condoms (Table 3).

Proportions of correct/incorrect answers to questions related to HIV/AIDS are summarized in Table 4. Almost half of the participants considered being physically fit as a way of not being infected with HIV and 29.0% reported that eating and sleeping well could protect against HIV infection. Those who considered that HIV positive individuals always feel very sick or present symptoms of the disease as soon as they get infected were 69.3% and 43.0% of participants, respectively. Beliefs that AIDS is a punishment for committing sins, that HIV was produced in a US laboratory, and that condom lubricants can contain HIV were reported by 38.2%, 67.2% and 32.4% of drug users surveyed, respectively.

**Table 4:** Correct knowledge about AIDS and modes of HIV transmission by previously HIV testing of 295 participants of the project "Assessing HIV Oral Testing of Brazilian Drug Users".

	HIV testing in life			p value
		
	No	Yes	Total	
The majority of people who transmit HIV looks sick	54.3	69.2	60.4	0.001***
Performing oral sex on someone brings risk of transmitting HIV	17.3	16.7	17.1	0.880
Staying physically fit is the best way to prevent HIV/AIDS	45.1	56.7	49.8	0.051*
Condom make sex completely safe	81.5	79.2	80.6	0.619
Take a shower after having sex decreased significantly the HIV transmission	61.3	71.7	65.5	0.066*
When a couple decides they'll ONLY have sex with each other they no longer need to use condoms	53.2	62.5	57.0	0.113
Most people exposed to HIV presents soon symptoms of being very sick	50.9	65.8	57.0	0.011**
Having few sexual partners, someone is effectively protected from HIV/AIDS	63.6	74.2	67.9	0.056*
Sharing toothbrushes can transmit HIV	38.7	52.5	44.4	0.020**
A person must have many different sexual partners to be at risk of HIV/AIDS	50.9	58.8	54.1	0.180
People who have HIV always feel very sick	27.8	35.0	30.7	0.186
Healthy people belonging to risk groups for AIDS should not donate blood	65.9	68.3	66.9	0.663
Is not risky share forks and spoons with a person who has HIV/AIDS	60.5	58.3	59.6	0.715
Eating and sleeping well protects a person against HIV/AIDS	67.6	75.8	71.0	0.128
It is more important to use condoms and clean needles in big cities than in small cities	45.7	63.3	52.9	0.003***
A negative HIV test result can occur even in people who have the virus	61.9	67.5	64.2	0.321
Coughing does not transmit HIV/AIDS	75.1	77.5	76.1	0.642
In anal sex (penis in anus), only the receptive partner can be infected by HIV	52.0	68.3	58.7	0.005***
The majority of cases of HIV/AIDS is caused by blood transfusions that occurred before 1984	30.1	29.2	29.7	0.870
Most people who have HIV know they have the disease	49.7	60.0	53.9	0.082*
People who donate blood are not at risk of get infected by HIV/AIDS	40.5	45.8	42.7	0.361
People do not get infected by HIV/AIDS if kiss someone's face or mouth (without tonge)	81.5	82.5	81.9	0.827
HIV can be transmitted by mosquitoes or cockroaches	74.0	70.8	72.7	0.551
AIDS is a punishment for committing sins	61.3	62.5	61.8	0.832
Anal sex is an alternative to vaginal sex in order to prevent HIV infection	66.5	83.3	73.4	0.001***
HIV can be transmitted through saliva	55.5	55.8	55.6	0.954
HIV was produced in a laboratory in the United States	30.6	35.8	32.8	0.351
HIV can pass through the pores of a condom	55.5	63.3	58.7	0.180
A person can be infected by HIV if an HIV-positive person spit on it	74.0	84.2	78.2	0.038**
Condom lubricant may contain HIV	65.9	70.0	67.6	0.461
It is not risky to use the same bathroom of person with HIV/AIDS	51.5	55.0	52.9	0.549

Rio de Janeiro, 2006.

* 0.10 ** 0.05 *** 0.01

The vast majority of participants (80.6%) reported correctly that condoms make sex safer (although, as shown, this does not correspond to actual safer practices). Almost 46% of individuals said that a person needs to have many different sexual partners to put him/her at risk of acquiring HIV infection and 32.1% believed by having fewer sexual partners, one is effectively protected against HIV. Almost half (41.3%) of the interviewees believed the AIDS virus could pass through the pores of a condom. About 36% of respondents were unaware that a negative HIV test could be found even in people who have the virus (as in the case of persons with recent infection, during the so-called "window period").

Almost half the interviewees (44.4%) believed HIV could be transmitted by saliva, and 55.6% said that sharing a toothbrush could transmit HIV. Most people knew that HIV could not be transmitted by mosquitoes and cockroaches, although over a quarter of respondents (27.3%) believed HIV transmission may occur through these putative vectors. Slightly more than half of the sample (52.9%) knew that using the same bathroom a person with HIV/AIDS had used presents no risk of acquiring HIV infection.

It should be noted that the item that produced the worst score, with only 17.1% of participants selecting the correct answer concerns transmission through oral sex. However, such a low ratio could reflect ambiguity in our phrasing (i.e., "performing oral sex on someone is risky for transmitting HIV"), which may not be the clearest way to refer to the possibility of the partner performing oral sex to transmit the virus to his/her partner who "receives" oral sex.

Notably, a significant difference in the proportion of correct/incorrect answers to some of the questions on HIV/AIDS was found between individuals who had been ever tested for HIV and those who had not. Those previously tested invariably presented a more accurate level of knowledge about AIDS and the ways HIV could be transmitted (Table 4). Such differences involve beliefs about the appearance of people with the virus, self-reported serostatus, transmission through anal sex, and potential transmission through saliva and the shared used of a toothbrush. The actual number of interviewees found to be HIV-infected was 3.7% (11/295) as defined by Orasure^® ^saliva.

More than half (52.5%) of the participants who had been tested previously answered correctly that HIV cannot be transmitted by *sharing a toothbrush *[italics for incorrect answers], but among those who had not been tested, the percentage of those who answered correctly was 38.7% (p = 0.020). A significant difference was found also with the item that stated that *anal sex could be an alternative to vaginal sex in order to prevent HIV infection*. Among those who had not been tested before, 66.5% answered this was not a valid alternative, but this percentage was significantly higher (83.3%) among individuals who had been tested before (p = 0.001). In the same way, those who were tested before were more likely (68.3%) to answer that in anal sex, both partners (active/passive or inserter/receiver) can become infected with the AIDS virus, compared to the untested individuals (52.0%) (p = 0.005).

Despite the evident associations between HIV testing and knowledge, it is not possible to discern their directionality, since, in the context of a cross-sectional study, it is not possible to say whether the very act of being tested for HIV - that according to Brazilian legislation must include pre and pos-test counseling - has positively influenced HIV/AIDS knowledge, or if having a better understanding of HIV/AIDS and a greater concern about personal vulnerability may foster test seeking behaviors.

**Figure 1:**
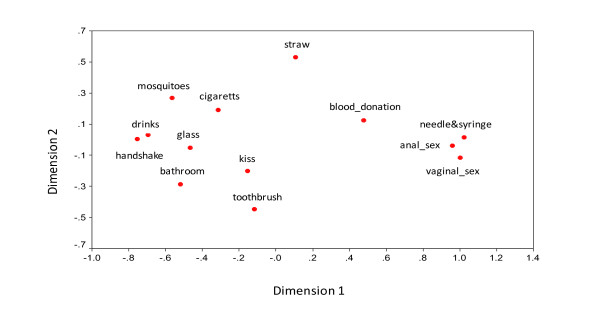
**Perceptual map of modes of HIV transmission. Rio de Janeiro, 2006**.

The perceptual map of the modes of HIV transmission was generated based on the responses of 108 drug users who participated in the pile sorting intervention. As depicted in Figure 1, items related to anal and vaginal sex and sharing syringes/needles were classified in a single spatial cluster, perhaps because these items were seen as effective means of HIV transmission by most of the participants (as well as by experts).

As mentioned earlier, greater distance between items on the map corresponds to conceptual dissociations - items related to *transmission by mosquitoes, sharing cigarettes, sharing drinks, using a common cup using the same toothbrush, social contact (e.g. shaking hands or a social kiss), as well as the sharing of public bathrooms *clustered in different areas from correct responses on modes of transmission (anal and vaginal sex and sharing syringes and needles), and were more widely separated from each other. This spatial dispersion may correspond to uncertainty among respondents as to whether such means are or are not effective ways of transmitting HIV.

The smaller the distance of an incorrect answer in relation to the cluster of items health experts identify as the riskiest behaviors for HIV (vaginal sex, anal sex, and the sharing needles and syringes), the greater the level of incorrect knowledge regarding this particular item. In this sense, participant beliefs about *blood donation *as risky represent the highest degree of erroneous knowledge, since most of individuals classified this event as a possible source of HIV infection to the donor. The Stress-1 was 0.095, indicating a good perceptual map fitness.

Comparing the responses of the interviewees obtained by the interview with results from the pile sort (i.e., information extracted from a visual stimulus), we observed agreement between these two methods in slightly more than half of respondents (58.3%) with respect to the putative *transmission of HIV through the shared use of public restrooms*. An agreement of 69.4% was found for *sharing toothbrushes*, and a somewhat lower proportion (62.9%) associated with *blood donation*, which was the item found in the multidimensional scaling to produce the lowest percentage of correct answers.

Although HIV transmission through *mosquito bites *produced the highest level of agreement between responses obtained by the two different methods, with 87.0% indicating this was not a way to acquire HIV infection, some doubts remain about this item because of the frequency of questions asked by participants about HIV transmission *through the bite of a mosquito *at the end of the pile sort activity (data not shown).

## Discussion

Our findings point to an increased level of HIV infection vulnerability among drug users compared to the general Brazilian population as found in population-based surveys [23], compounded by the high frequency of risk behaviors among drug users as found in this study as well as reported in the Brazilian and international HIV/AIDS literature [19].

Knowledge of ways of transmission, risk perception and attitudes and practices of individuals and groups related to sexual behavior or drug use are central elements in defining individual vulnerability. These aspects are in synergy with each other and closely influence both individual behavior and behavior changes [24].

The high prevalence of alcohol and illicit drug use in our sample reflects a polydrug use pattern (that is, users of many different substances in various combinations), although the study design (cross-sectional) and the structure of questionnaire did not allow us to assess each of the combinations, in each specific context of use and different periods of participants life trajectories. At the time, this study was carried out (2006-7), the use of crack was relatively low in Rio de Janeiro, in frank contrast with subsequent studies carried out with the same population and in the same setting, including studies showing a crack cocaine prevalence of 68% [25]. However, such comparison must be viewed with caution due to the relatively small convenience sample, in comparison to a much larger study using Respondent Driven Sampling carried out in the second quarter of 2009, and because the period of drug consumption analyzed is different in these studies. Several studies highlight a significant increase in the use of crack in recent years, in various Brazilian localities [26,27].

As evidenced by previous research [28], injection of liquefied cocaine powder appears to be a relatively rare event in Brazil, especially in Rio de Janeiro, where it was never common among street drug users, in comparison with some southern Brazilian cities [29]. Heroin was reported by a very small number of respondents, corroborating previous findings documenting its infrequent use in the Rio de Janeiro drug scene [30].

Our findings highlight the need to implement interventions targeted to drug users, including people who misuse alcohol, not only because of the risks and harms associated with drug use itself (e.g., dependency, overdose), but also because of the adverse influence of substance use, abuse and dependence on the adoption of safer behaviors. Such interrelationships tend to be complex and comprise recursive interrelationships which cannot be assessed with the necessary depth by cross-sectional studies and are only partially explored by other epidemiologic design as discussed by Fortenberry et al. (1997) [31], in their landmark study on sex under the influence using diaries.

Major gaps still seem to exist in knowledge among drug users about the ways HIV may be transmitted/acquired. Although 80% of respondents in this study responded correctly that condoms protect against HIV during sexual intercourse, this percentage was lower than the one found in studies conducted with the general Brazilian population, where 90% of respondents in 2005 answered correctly [12]. Such differences may be partially secondary to the lower educational level of our interviewees vis-à-vis the overall standards of the Brazilian general population. Ferreira et al. [12] clearly documented the poor knowledge on HIV/AIDS among people with lower educational levels compared to those with a college degree in a representative sample of Brazil urban population. Notwithstanding the impossibility to disentangle the specific role of social and behavioral variables, the synergistic influence of them on less than optimal knowledge on HIV/AIDS among impoverished drug users speak in favor of comprehensive preventative initiatives tailored to the specific needs of underserved people, and among them, among those people who are misuse substances [18]. One should remember here that over 40% of participants believed HIV could pass through the pores of a condom.

In this population, there are still beliefs that a nice appearance and physical fitness are associated with the absence of HIV infection and a high proportion of interviewees reported that people who are HIV-positive look sick and that their symptoms appear immediately after they get infected. This perception could be accurate in countries with uneven access to antiretrovirals (ARVs), but this is certainly not the case of Brazil, the middle-income country with the oldest (mandated by federal legislation as of 1996) and most comprehensive (considering the number of people estimated to be living with HIV) program of universal access to ARVs, worldwide [32].

These findings may be associated with prejudice directed to people living with HIV, as portrayed in the media some years ago and still stereotyped by many people, especially those less informed about the progress of HIV medicine in recent years and the ample access to treatment in Brazil.

Individuals who had been tested before for HIV showed a better level of HIV/AIDS knowledge compared with those who had never been tested. Although the causal directionality of this association cannot be discerned by our study methods, and should be viewed with caution in a study based on a convenience sample, test seeking behaviors, actual HIV testing, sound knowledge on HIV/AIDS, as well as appropriate counseling probably comprises a mutually reinforcing cycle pointing to healthier habits and attitudes. To the degree that HIV misinformation is a barrier to HIV testing it constitutes a critical issue that must be addressed in efforts to scale up emergent "find, test, and treat" models of HIV intervention. Misinformation about HIV (including misperceptions of personal risk) may be an important factor contributing to the significant number of individuals who are HIV positive but are not aware of their serostatus because they have never been tested.

There may be some value in attempting to determine the sources of HIV misinformation among drug users in Rio de Janeiro. It may well be that HIV/AIDS prevention efforts inadvertently contributed to participant beliefs about the high level of safety of having fewer sexual partners. In stressing the risk of having multiple partners, prevention efforts may have sent the message that having a small number of partners is safe and hence does not require an individual to use condoms to prevent infection.

It is harder to discern the source of misinformation about HIV passing through the pores of a condom since we did not assess the worldviews and religious practices of each interviewee with the necessary depth. However, they may have their origin in exposure to anecdotal information disseminated over the years by conservative sectors of the Catholic Church in Brazil, and fully available in religious newspapers and the internet. The recent statement made by Pope Benedict XVI himself [33] seems to be pivotal in rectifying such misinformation, unfortunately common in the largest Catholic country in the world.

Beliefs about mosquitoes seem to have their roots in the centenary public health campaigns concerning mosquitoes as disease vectors (e.g., dengue fever), which has been a significant health problem in Brazil since the early 1900s.

Finally, there is the issue of appearance and fitness as sources for determining HIV status and protecting against infection. These ideas may reflect contemporary emphasis on leading healthy lifestyles. Such lifestyles are clearly beneficial in terms of cardiovascular health and prevention/management of metabolic disorders, however they can be misunderstood as giving full protection to hypothetically "100% healthy people" against any disease. This can take place despite the very clear warnings issued by recent public health campaigns in Brazil that exercising is a key component of a healthy lifestyle, but does not exempt young, healthy people to protect themselves, for instance vaccinating themselves against influenza A. Believing that healthy looking people don't have HIV can make a person less likely to get tested once they don't feel sick, and also probably less likely to ask their partner about their status, because they think they look healthy so they must be safe.

While there may well be other influences for each of the items discussed, it is clear that individuals construct their understandings of healthy and unhealthy behaviors from their cultural milieu and that this pattern occurs among marginalized drug users as much as among individuals who embrace mainstream behavior sets. Consequently, public health efforts must be particularly careful in the selection and phrasing of health promotion messages, as such messages may be unintended sources of consequential misinformation [34].

Our findings from the perceptual map may help to discern three levels of cognitive dissonance and inform prevention targeting this population.

First, beyond a core of consistent and practically consensual responses, there are different intensities of dissonance between the perceptions of drug users and what science recognizes as safe behaviors. Broad and targeted campaigns, consequently, should emphasize blood donation as a risk-free event in the Brazilian context, since it is performed in accredited sites that invariably use sterile equipment [35].

Second, different research strategies for data collection on HIV knowledge should be used in light of the fact that we found differences in the data collected using different research methods. This finding affirms the idea that the way(s) one obtains information may bias the proper assessment of HIV/AIDS knowledge.

Finally, despite fairly consistent and accurate information regarding items that express key high-risk behaviors (e.g. unprotected vaginal and anal sex), such perceptions should not be understood as conducive to the consistent adoption of safer sexual practices, as has been frequently observed by different studies evaluating the difficulties to translate sound information into concrete behavioral change in multiple areas of public health (smoking prevention, traffic accidents associated with alcohol misuse and/or no usage of seatbelts, etc...).

Our findings speak in favor of targeted prevention initiatives, and argue against accepting the generalized perception that Brazilians have a sufficiently high level of information after three decades of sustained preventive efforts that such initiatives can be substantially reduced or limited to special occasions. Our findings reinforce the need to tailor interventions to hard-to-reach, highly at risk populations, such as people who misuse drugs and/or impoverished social strata, as these may be people who have limited and contradictory information about HIV/AIDS and are still engaged in high-risk behaviors.

The present study has certain limitations that need to be taken into account. We accessed a small convenience sample, using a semi-quantitative method (multidimensional scaling). Notwithstanding, considering the low prevalence of heavy users in the general population [36] and the hidden nature of such populations, it would be dangerous to simply infer from data obtained from large population-based studies assessing the knowledge, behaviors and attitudes of the general Brazilian population that prevention is passé. As discussed by a recent paper by our group [19], some segments of the population have been disproportionately facing risks of acquiring HIV and other STIs, despite three decades of concerted and continuous initiatives and a successful partnership between different levels of government and civil society. Reaching such populations with messages informed by studies like the one reported here, as well as fostering HIV testing and counseling among them should remain key topics in the Brazilian health policy agenda. Messages that directly address specific items of misinformation and make use of insights from learning science [37-39] about how to best frame public health communications, is a critical issue for ongoing HIV/AIDS prevention in Brazil.

## Competing interests

The authors declare that they have no competing interests.

## Authors' contributions

NB was responsible for data analysis and writing the manuscript, as part of her MPH dissertation, mentored by FIB and co-mentored by CMFPS. SC was the PI of the original study and MS the co-PI. All authors participated of the field work, reviewed the analyses and drafted the article. All authors have given final approval for this version of the manuscript.
